# Recent advances in research on common targets of neurological and sex hormonal influences on asthma

**DOI:** 10.1002/clt2.70022

**Published:** 2025-01-12

**Authors:** Wenting Zhou, Huan Chen, Xinyu Chen, Jing Gao, Wenting Ji

**Affiliations:** ^1^ China Chengdu University of Traditional Chinese Medicine Chengdu Sichuan China

**Keywords:** asthma, nerve, neuronal regulation, sex hormone

## Abstract

**Background:**

Asthma is currently one of the most common of respiratory diseases, severely affecting the lives of patients. With the in‐depth study of the role of the nervous system and sex hormones on the development of asthma, it has been found that the nervous system and sex hormones are related to each other in the pathway of asthma.

**Objective:**

To investigate the effects of sex hormones and the nervous system on the development of asthma.

**Methods:**

In this review, we searched for a large number of relevant literature to elucidate the unique mechanisms of sex hormones and the nervous system on asthma development, and summarized several common targets in the pathways of sex hormones and the nervous system on asthma.

**Conclusion:**

We summarize several common important targets in the pathways of action of sex hormones and the nervous system in asthma, provide new directions and ideas for asthma treatment, and discuss current therapeutic limitations and future possibilities. Finally, the article predicts future applications of several important targets in asthma therapy.

## 1 INTRODUCTION

Asthma is a common chronic inflammatory disease of the airways that can lead to variable or even constant airflow limitation. The main symptoms are dyspnoea, shortness of breath, chronic cough and chest tightness.^1^ The prevalence of asthma can be as high as 18% in some countries, with a total of more than 339 million asthmatics worldwide.^2^ Among respiratory diseases, asthma affects about one third of the world population. It is estimated that nearly 2.5 million people die each year from severe exacerbations of asthma.^3^, ^4^ But asthma‐related deaths are thought to be largely preventable, especially among children and adolescents.^5^ According to the latest data from the Centres for Disease Control and Prevention (https://www.cdc.gov/), adults are nearly five times more likely to die from asthma than children. Asthma deaths are highest in the 65‐and‐over age group compared with other age groups and are higher among women. Asthma has a serious impact on the mental and physical health of people, leading to reduced quality of life, reduced learning productivity and limited physical activity. Severe asthma is difficult to treat and requires high doses of steroids or other medications to control the disease or the disease remains uncontrolled despite such treatment.^6^ Therefore, the understanding of ways to effectively treat asthma has become an essential goal of asthma control in the last few years.

The effect of sex hormones on asthma has been a topic of great interest to scholars.^2^, ^7^, ^8^ In recent years, some researchers have also suggested that the nervous system is also inextricably linked to asthma.^9^, ^10^ Limited evidence also suggests that neurons and sex hormones can interact and regulate each other to some extent. Neurons can be involved in the production of sex hormones,^11^ and sex hormones regulate neuronal excitability,^12^, ^13^ plasticity^14^, ^15^ and so on. But the effects of the interaction of both neurons and sex hormones on a number of diseases, such as allergic rhinitis, asthma and other allergic diseases, are poorly understood and could potentially open up new ideas for treating these diseases.

**FIGURE 1:**
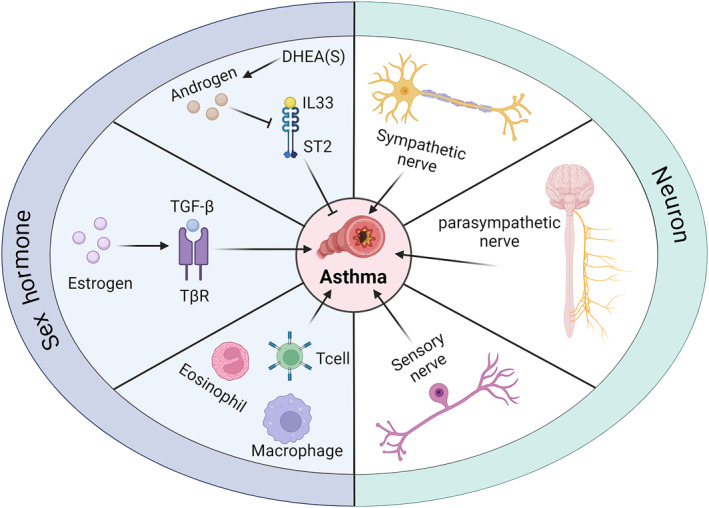
Effects of sex hormones and nerves on the pathogenesis of asthma. This picture was created by biorender.

In this regard, we propose that there may be a co‐regulation between sex hormones and neurons in the pathogenesis of asthma. This paper systematically organizes the interactions and effects of asthma with sex hormones and the nervous system. Through the summary and organization of previous studies, several common and important targets in the pathways of action of sex hormones and the nervous system affecting asthma have been identified, which act on the development of asthma. The sex hormones and the nervous system work together on these targets to lead to the development of asthma. This helps to develop new target therapies. By inhibiting the action of these targets not only can we directly block their direct effect on asthma but also to a certain extent can weaken the influence of the nervous system and sex hormones on asthma, playing a positive feedback effect, with a view to providing more options for asthma treatment (Figure 1).

## 2 ASTHMA AND SEX HORMONES

### 2.1 Gender differences in asthma prevalence

There are significant gender differences in the prevalence and severity of asthma. According to the most recent data from the Centres for Disease Control and Prevention, the national prevalence of asthma in the general population of the United States is approximately 7.7%, with a higher prevalence in female adults (9.7%) than in male adults (6.2%) (Centers for Disease Control and Prevention (cdc.gov)). Amber H Sinclair and her team gathered data from electronic administrative data, patient surveys and hard‐copy chart reviews to illustrate gender differences in asthma, ultimately finding that women suffered from more asthma symptoms, severity, restriction of activities, use of asthma reliever medication, and higher rates of asthma visits, as compared to men.^16^ Graphs of Global Health Data Exchange 2018 data show changes in asthma prevalence in men and women in developed countries, demonstrating the role of sex hormones.^2^ A growing amount of evidence suggests that sex hormones work central to these sex differences and interact directly with multiple key cells involved in the pathogenesis of asthma.^17^

### 2.2 Sex hormone modulation of asthma pathogenesis

**FIGURE 2:**
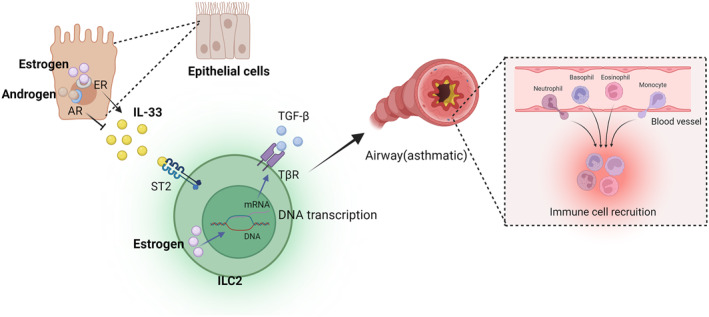
Oestrogen releases IL‐33 upon binding to the receptor in epithelial cells and acts on the ST2 receptor on ILC2, causing airway inflammation. Androgens play an inhibitory role in this process. Oestrogen can influence the development of asthma by regulating the expression of transforming growth factor‐β (TGF‐β) receptors. This picture was created by biorender.

Sex steroid signalling in asthma pathogenesis virtually affects all organ systems, including the immune cell system. During the last decades, there has been a growing realisation that chemokines and cytokines play an important role in the underlying mechanisms that sustain asthma progress in the long term (Figure 2).

#### 2.2.1 Immune cell

Although the majority of airway damage observed in asthma is caused by sustained inflammation involving a range of immune cell components, it is still widely recognised that it is the T cells have a central function in asthma pathophysiology.^18^ In asthma pathology, evidence on the correlations between sex hormones and the immune reactions has been published for more than 20 years.^19^, ^20^, ^21^ Testosterone or androgen receptor (AR) is generally considered to have an effect on overall immunosuppression.^22^, ^23^ Androgens negatively regulate asthma inflammation by targeting ILC2 and Th2 cells to weaken IL‐17A‐mediated responses and leukotriene biosynthetic pathways.^24^ Ghadeer AbdulHussain's research group determined the ability of lymphocytes to produce cytokine patterns using multiculture and enzyme‐linked immunosorbent assays in women with a history of recurrent spontaneous abortions, and found that production of the Th2 cytokine IL‐6 was upregulated under the influence of oestrogen. This combination of inhibition of the Th1 cytokines IL‐2 and IFN‐γ and concomitant enhancement of the production of the Th2 cytokine IL‐6 suggests that oestrogens are able to bias cytokine production in favour of Th2 dominance, with implications for Th2‐type immune disorders.^25^

#### 2.2.2 DHEA‐S

DHEA‐S and its metabolite dehydroepiandrosterone (DHEA) are metabolites of cholesterol which are further metabolised to androgens. There is evidence that levels of DHEA and DHEA‐S decrease in asthma patients during both stable and deteriorating periods of asthma.^26^ Cynthia J. Koziol‐White and her team's study evaluated the effects of DHEA‐S on human airway smooth muscle cells migration and human peripheral blood neutrophil.^27^ The results showed that inflammation of allergic airways induced by ovalbumin was significantly weakened by DHEA‐S in mice, and that DHEA‐S attenuated ASM and neutrophil migration. This suggests that DHEA‐S can not only inhibit the secretion of inflammatory mediators but also inhibit the migration of myocytes and immune cells. This may help to treat airway diseases such as chronic obstructive pulmonary disease (COPD) and asthma as a new therapeutic approach for airway diseases.

#### 2.2.3 TGFβ

Transforming growth factor‐β (TGFβ) is a poly‐functional growth factor that participates in the regulation of key events in development, disease and tissue repair. Airway remodelling is thought to involve one or more isoforms of transforming growth factor.^28^ In an animal model of asthma, increased TGF‐β1 expression and bronchial/peribronchial ASM growth occur concurrently, and the use of drugs targeting TGF‐β can prevent or reverse airway remodelling.^29^ Shyamal K. Roy found that gonadotropins and ovarian steroid hormones positively regulate TGF receptor mRNA expression in hamster ovaries by analysing changes in the mRNA expression pattern of TβRI and TβRII in mouse ovaries during the reproductive cycle.^30^ Yuhao Gao and his team found that bone‐derived macrophages from ovariectomised mice exhibited reduced TGF‐β1 mRNA, which was rescued by exogenous treatment with oestrogen.^31^ These evidences suggest that oestrogen may promote tissue remodelling in allergic inflammation by inducing TGF‐β.

## 3 NEURONAL REGULATION OF ASTHMA

**FIGURE 3:**
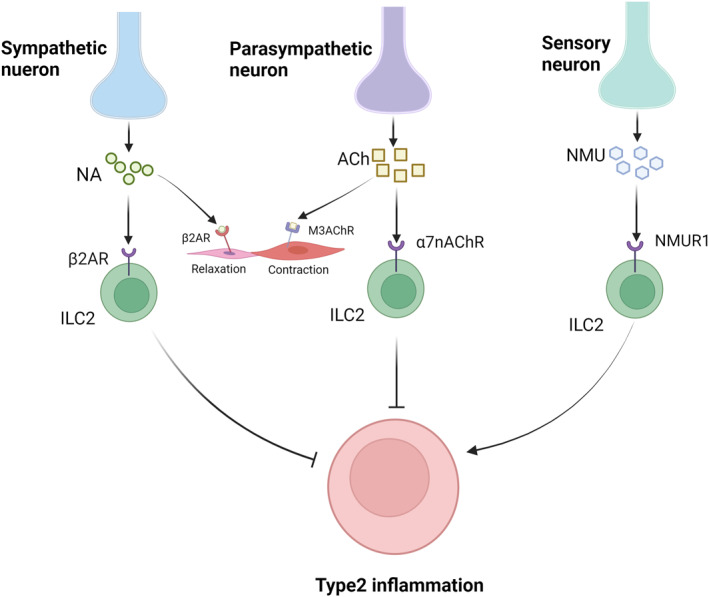
Sympathetic, parasympathetic, and sensory nerves release different neurotransmitters, including NA, Ach, and NMU, which act on ILC2, which in turn affects the inflammatory response. This picture was created by biorender.

Neurons make an indelible difference in the pathogenesis of asthma (Figure 3). Neurons release neurotransmitters that directly regulate airway bronchial muscle contraction and diastole to control asthma. However, in recent years, research on neuroimmune crosstalk has received increasing attention, and many studies have shown that neuronal regulation is possible through immune cell action.^32^, ^33^, ^34^ Hiroki Kabata and David Artis propose that the immune system and neurons demonstrate two‐way interactions and play significant roles in infection, inflammation and tissue haemostasis. Neuron‐derived neurotransmitters and neuropeptides regulate immune cell function, while inflammatory mediators produced by immune cells enhance neuronal activation.^32^ Therefore, in the pathogenesis of asthma, neurons may also act through this mechanism.

### 3.1 Neuronal regulation of asthma by sympathetic nerves

Norepinephrine and epinephrine released by sympathetic neurons can be involved in bronchial muscle relaxation, and are therefore commonly used clinically as bronchodilators in the treatment of asthma and COPD.^35^ A variety of immune cells in vivo express norepinephrine and adrenergic receptors, α‐adrenergic receptors and β‐adrenergic receptors and are directly stimulated by catecholamines. Epinephrine and norepinephrine inhibit ILC2‐less mediated type 2 inflammation primarily through agonism of β‐adrenergic receptors located on ILC2 and induce smooth muscle cell relaxation.^36^ Saya Moriyama and her team demonstrated that mouse ILC2 expresses β2 adrenergic receptors (β2AR) and co‐localises with adrenergic neurons in the gut. β2AR deficiency leads to ILC2 overresponsiveness and T2 inflammation in lung tissues and intestinal. In contrast, β2AR agonism is associated with impaired ILC2 responses and reduced inflammation in vivo, which promotes β2AR agonist treatment of inflammation.^37^ Sympathetic neuron‐derived Noradrenaline (NA) inhibits the β2AR by agonising the β2AR located on the ILC2.

### 3.2 Neuronal regulation of asthma by parasympathetic nerves

The airways are intensively innervated by sensory and parasympathetic nerves that regulate airway tone and cause narrow airway constriction. The vagus nerve contains parasympathetic fibres. Parasympathetic nerves transmit the airways from the brain and autonomously control bronchoconstriction. Furthermore, parasympathetic nerves activate M3 muscarinic receptors on smooth muscle of the airway by releasing acetylcholine (ACh) to stimulate bronchoconstriction and induce mucus secretion and bronchial vasodilatation.^38^ A recent study suggests that ILC2 may be regulated by cholinergic signalling, suggesting that acetylcholine can modulate type 2 immune inflammatory responses. This effect is elicited by acetylcholine via α7nAChR, an α7nAChR agonist that directly inhibits ILC2. Acetylcholine acts as an anti‐inflammatory via α7AChR inhibition of ILC2, inhibiting macrophage, basophil, and mast cell activation, thereby reducing type 2 inflammation in airway inflammation.^39^, ^40^

### 3.3 Neuronal regulation of asthma by sensory nerves

Sensory neurons contain a wide variety of neuropeptides, such as substance p and substance crp. Allergens can stimulate the induction of the release of these neuropeptides. Studies have shown that sputum levels of substance P are elevated in patients with asthma and chronic bronchitis, and that substance p is associated with airway obstruction.^41^ Dimitri Tränkner and his team used a mouse model of acute asthma to demonstrate that a subset of sensory neurons mediate respiratory over‐responsiveness, and they genetically ablated certain populations of sensory neurons, with the end result that these animals do not develop ovalbumin‐dependent airway hyper‐responsiveness.^42^ The results suggest that sensory nerve blockade ameliorated respiratory inflammation type 2. In addition, neuropeptides derived from sensory neurons activated ILC2 and Th2 cells, resulting in enhanced T2 inflammation. IL‐5 activated sensory neuron terminals and caused further release of Vasoactive Intestinal Polypeptide. Neuromedin U (NMU) also directly activated ILC2, leading to T2 inflammation.^43^ Instead, interrupting this neuroimmune interaction with QX‐314, a voltage‐gated sodium channel blocker, blocks the activation of sensory neurons, which may inhibit neuropeptide release and attenuate type 2 inflammation, thus revealing a potential new therapeutic strategy for asthma.

## 4 EFFECTS OF SEX HORMONE‐NEURON INTERACTIONS ON ASTHMA

As mentioned above, sex hormones and neurons each make a difference in the pathogenesis of asthma; however, there is relatively little research on whether there is some association between the sex hormones and the nervous system that affects the development of asthma. However, this idea suggests a new avenue for the future treatment of asthma, which is to inhibit a common target of both actions in order to weaken the pathways of action of the sex hormones and nervous system on how asthma develops, thus providing a positive feedback to suppress asthma symptoms. Therefore, here we ask whether there is some role or some target between sex hormones and neurons to co‐regulate asthma pathogenesis. Before doing so, we first understand the interaction between neurons and sex hormones.

### 4.1 Sex hormone action on neurons

Numerous studies have shown that gonadal steroids can regulate reproductive or neuroendocrine effects, participate in the formation of brain developmental processes, and are associated with higher brain functions such as cognition, mood, and memory. The question of how the relevant sex hormones affecting the development of asthma act on neurons is one that deserves to be explored and studied in depth.

#### 4.1.1 Protective effects of sex hormones on neurons

Both oestrogens and androgens are well‐documented as having neuroprotective properties. In vitro and ex vivo experimental studies confirm that oestrogen protects neurons from glutamate, glucose deprivation, and beta‐amyloid peptide‐induced injury and reduces infarct size after middle cerebral artery occlusion.^44^, ^45^, ^46^ Testosterone also significantly reduces beta‐amyloid neurotoxicity by a rapid oestrogen‐independent mechanism.^47^

We know that DHEA is a precursor hormone for several bioactive sex steroids, such as oestradiol and testosterone. We have mentioned above that dehydroepiandrosterone‐3‐sulfate (DHEAS) and DHEA can restrain the secretion of inflammatory mediators and attenuate allergic airway inflammation. However, several studies have demonstrated the neuroprotective effects of DHEAS and DHEA under different experimental conditions, including spinal cord injury, neurodegenerative disease, ischaemia, glutamate excitotoxicity and traumatic brain injury models.^48^ V. G. Kimonides and his team found that DHEA(S) prevented or reduced the neurotoxic effects of the glutamate agonist N‐methyl‐d‐aspartate (NMDA) in the hippocampus, both in vitro and in vivo, further confirming the protective effect of DHEA(S).^49^

We previously mentioned that gonadotropins and ovarian steroid hormones can positively regulate TGF receptor mRNA expression in hamster ovaries and induce TGF‐β to promote tissue remodelling in allergic inflammation. And studies have shown that TGF‐β also protects neurons from diverse injuries. Transforming growth factor‐β 1, 2 and 3 are expressed in the brain of adults. Transforming growth factor‐β promotes the recovery of motor and dopaminergic nerves in the rat spinal cord in in vitro experiments. The expression of TGF‐ β1 is increased in astrocytes and microglia in animal models of cerebral ischaemia, and the expression of TGF‐ β2 is increased in activated astrocytes in human neurodegenerative diseases. Therefore, a lot of studies have reported that TGF‐β is a potent neuroprotective agent that can influence central neurodegenerative diseases.^50^ This further supports the idea that sex hormones play a protective role in neurons while regulating the development of asthma.

#### 4.1.2 Neuronal plasticity of sex hormones

The brain produces sex hormones, and there is evidence that such brain‐derived gonadotropins may play a significant role in synaptic remodelling. A Parducz's selection of letrozole, an aromatase inhibitor used to inhibit the synthesis of oestradiol,^15^ for addition to adult hippocampal neuronal media resulted in a significant reduction in the amount of oestrogen synthesised. The number of spinal synapses was then analysed under electron microscopy using an unbiased somatotopic approach compared with the presynaptic. It was found that coming oestradiol deficiency resulted in a key reduction in the quantity of spinal synapses and synaptic spines. This confirms that oestradiol plays a regulatory role and is essential for synapse formation.

There has been evidence in the last decade that TGF‐β ‐ a transforming growth factor thought to be involved in airway remodelling and regulated by oestrogen—may be an important regulator involved in neuronal plasticity. Luan Pereira Diniz and his team found in vivo and in vitro the formation of inhibitory synapses in cortical neurons induced by TGF‐β in conditioned media from human and mouse astrocytes. TGF‐β1 induces localisation as well as cluster formation of synaptic adhesion protein at inhibitory postsynaptic terminals through activation of CaM kinase II and glutamatergic activity.^51^ In addition, intracerebroventricular injection of TGF‐β1 resulted in an increase in the number of inhibitory synapses in the cerebral cortex. Furthermore, they found that both human astrocytes and mice were able to induce excitatory and inhibitory synapse formation by secreting TGF‐β1.^52^ This confirms that TGF‐β has a regulatory role and is essential for synapse formation, and that oestrogens, which regulate mRNA expression of TGF‐β, are inextricably linked to this role. The neurosteroid dehydroandrosterone (DHEA) and its sulphate (DHEAS) also enhance neuronal plasticity. Existing studies of human brain development support the possible involvement of DHEA in protecting cortical plasticity in cognitively controlled brain networks, and molecular studies also imply that DHEA plays a key role in enhancing brain plasticity.^53^

#### 4.1.3 Neuronal excitability regulated by sex hormones

In addition to its synaptic remodelling effects, oestrogen increases neuronal excitability. There is evidence that oestradiol increases the number of dendritic spines of CA1 pyramidal neurons in the hippocampus of female rats, and increases the production of mRNA for NMDA receptors and the density of excitatory NMDA receptors.^13^ This result led to enhanced calcium entry mediating NMDA and more stimulus input to pyramidal cells, causing enhanced excitation.

B Carette and P Poulain observed excitatory effects when DHEA, its pregnenolone sulphate and sulphate (DHEAS) were applied to neurons in the anterior region of the optic septum by iontophoresis or pressure.^54^ As a potential signalling molecule for neocortical organisation during brain development, DHEA or DHEAS can modulate neuronal function by regulating the release of a variety of neurotransmitters such as gamma aminobutyric acid, glutamate, acetylcholine, and others, and these modulations may lead to important changes in neuronal excitability.^55^ Granule cells (GCs) are the most abundant granulomatous neurons in the cortex of the cerebellum, and Ana P. B. Araujo showed by immunocytochemical analyses of pre‐synaptic and post‐synaptic proteins that a 100% increase in the number of glutamatergic excitatory synapses resulted from the treatment of GC cultures with TGF‐β.^56^ This suggests that TGF‐β has a important function in the cerebellum by regulating the formation of excitatory synapses between granule neurons and thus regulating neuronal excitation. TGF‐β1 has been shown to induce a long‐term rise in neuronal excitability in the sea rabbit. Application of TGF‐β1 (1 ng/mL) to separated sensory neurons for 6 h revealed that exposure to TGF‐β1 resulted in a 210% rise in excitability tested 24 h after application and a 115% rise in excitability tested 48 h after application. Moreover, TGF‐β1 treatment reduced the stimulation threshold of sensory neurons by 30% at both the 24 and 48 h time points.^57^ Jeannie Chin also repeated the above manipulation and similarly found that neuronal excitability increased by 238 ± 30% (mean ± SE) of baseline after 24 h.^58^ This further supports that TGF‐β has a modulating effect on neuronal excitability and acts as a positive regulatory control. It further reveals that sex hormones regulate asthmatic regression while not only protecting neurons and modulating neuronal plasticity but also modulating neuronal excitability.

### 4.2 Neuronal actions on sex hormones

#### 4.2.1 Widespread presence of sex hormone‐related receptors on neurons

Within neurons, sex hormone receptors are present in the nucleus and also located near the membrane, associated with presynaptic terminals, mitochondria, spinal columns, and postsynaptic densities. Sex hormone receptors have been found in almost every region of the nervous system including the hypothalamus, amygdala, and hippocampus.^58^, ^59^ It has been calculated that 100% of oestrogen in women is synthesised from DHEA and DHEAS. Oestrogens synthesised from DHEA and DHEAS can act by targeting sex hormone receptors on neurons. We know that mRNA expression of TGF‐βRII and TGF‐βRI is regulated by gonadotropins and ovarian steroid hormones.^60^ It has been demonstrated that TβRI and TβRII are widely distributed in neurons, astrocytes, oligodendrocytes, microglial cells, and brain endothelial cells,^61^ and that they bind to TGF‐β to exert neurological effects such as modulation of synaptic plasticity and neuronal excitability.

#### 4.2.2 Involvement of neurons in sex hormone production

Both astrocytes and neurons produce large amounts of 17β‐oestradiol in both male and female brains.^11^ 17β‐oestradiol is a steroid hormone that can be produced in the brain from androgen precursors by the action of aromatase. Aromatase activity in the brain has now been confirmed by assays such as enzyme activity measurements, protein expression, mRNA expression gene transcription and radiolabelled inhibitor binding that produces positron emission tomography images, and is present in many brain regions.^59^ In addition to this, the synthesis of certain sex steroid precursors, DHEA and DHEAS, which inhibit allergic airway inflammation, is also neurologically related. DHEA is mainly derived from the adrenal glands but can also be synthesised in the gonads (10%–20%).^62^, ^63^ It is now suggested that DHEA is likely to be synthesised in the central nervous system (CNS). Concentrations of DHEA have been found to be higher than plasma concentrations in the brain of both humans and mice^64^, ^65^ and remain high in the brain after adrenalectomy and gonadectomy in rats.^66^ Astrocytes have been shown to produce pregnenolone, progesterone, DHEA, and others.^67^ We know that the biosynthesis of dehydroepiandrosterone sulphate (DHEAS) is catalysed by hydroxysteroid sulphotransferase (HST), and the group of D Beaujean investigated the distribution of HST‐like immunoreactivity in the CNS of frogs and demonstrated for the first time that the biosynthesis of the neuroactive steroid DHEAS occurs in the CNS of non‐mammalian vertebrates.^68^ These pieces of evidence support the local synthesis of DHEA and DHEAS in the brain.

### 4.3 Common targets of nervous system and sex hormone action in asthma

**FIGURE 4:**
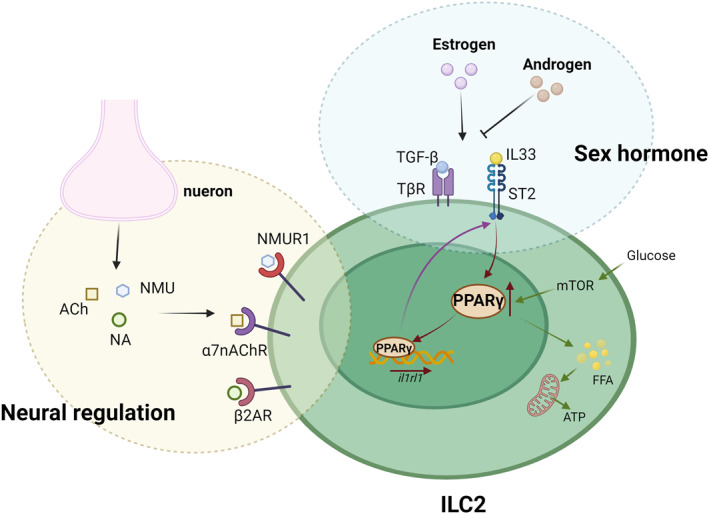
Sex hormones and neurons act together on different receptors on ILC2 to modulate the inflammatory response to regulate the development of asthma. PPARγ can affect airway inflammation by modulating the expression and metabolic response of receptors on ILC2. This picture was created by biorender.

**FIGURE 5:**
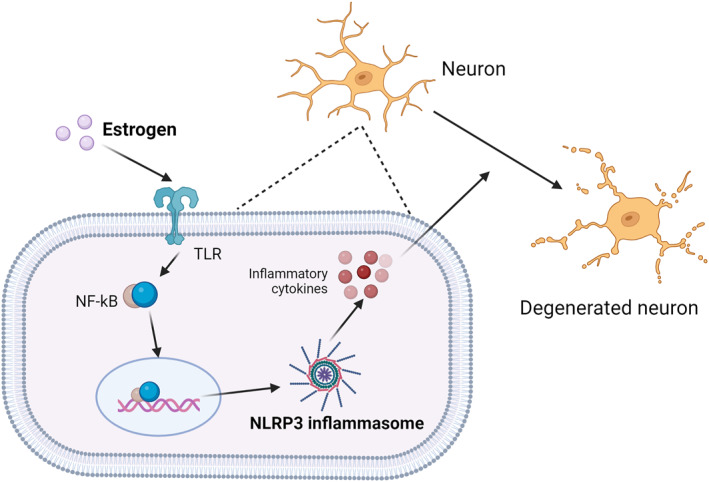
Oestrogen promotes the release of NLRP3 vesicles, leading to further release of inflammatory cytokines. These inflammatory cytokines in turn can further promote neuronal senescence and death. This picture was created by biorender.

As can be seen above, both the nervous system and sex hormones act in asthma, and the nervous system and sex hormones can interact with each other to some extent, and in this regard, we asked whether the nervous system and sex hormones act together on a certain target, thus affecting the pathogenesis of asthma. By reviewing a large body of literature, we identified a role for NLRP3 inflammatory vesicles, peroxisome proliferator‐activated receptor γ (PPARγ), and ILC2s in this regard (Figures 4 and 5).

#### 4.3.1 ILC2s

Group 2 innate lymphocytes (ILC2s) are a new type of non‐B, non‐T lymphocytes that produce large amounts of pro‐inflammatory and regulatory cytokines in response to inflammation, tumours, local injury or pathogen infection. ILC2s are considered to be the main initiator of airway inflammation,^69^ and many research groups have clearly demonstrated that ILC2s are increased in blood and sputum in asthmatics. ILC2 is closely related to the CNS. Glial cell line‐derived neurotrophic factors are expressed only by neurons, whereas these cytokines are expressed in CNS‐resident ILC2s, which may indicate communication between neurons and the ILC2.^70^ ILC2 expresses a variety of neurotransmitter receptors, such as the nerve growth hormone U receptor Neuromedin U Receptor 1 (NMUR1) and vasoactive intestinal peptide receptor 2, which mediate the connection between the peripheral nervous system and ILC2.^71^ Neurotransmitters secreted by neurons bind to these receptors to modulate ILC2, resulting in a series of responses. NMU secreted by neurons positively regulates the activation, proliferation, and cytokine production of ILC2s via NMUR1, thereby providing rapid tissue protection against helminthic infections.^72^ ILC2 also plays an important role in certain neurological diseases. For example, cerebral ischaemia mobilizes ILC2s to inhibit neuroinflammation and brain damage.^73^ In neuromyelitis optica spectrum disorder, a severe CNS autoimmune disease, ILC2 has a beneficial role.^74^ Depletion of ILC2 can lead to an increase in the volume of CNS lesions, decreased CNS glucose metabolism, and enhanced astrocyte damage and demyelination. We have previously mentioned that in the nervous system, sympathetic, parasympathetic and sensory nerves all act on ILC2s through a series of pathways to elicit a type 2 inflammatory response that triggers asthma. Adrenaline released from sympathetic neurons can agonize the β2AR on ILC2, thereby inhibiting ILC2‐mediated type 2 inflammation. Parasympathetic release of acetylcholine agonizes the α7nAChR which in turn inhibits ILC2 and acts as an anti‐inflammatory agent. Sensory neurons are induced to release neuropeptides after allergen stimulation, and neuropeptides activate ILC2 to enhance type 2 inflammation. In addition, Yingjiu Cao's group suggests that neuronal signalling has emerged as a key regulator of class 2 innate lymphocytes (ILC2), which modulate tissue haemostasis and allergic inflammation. They gave dopamine topically and found that it attenuated allergen‐induced ILC2 responses and airway inflammation.^75^ Further studies have shown that sex hormones also act on ILC2s to influence asthma pathogenesis. JY Cephus and his team found that in patients suffering from moderate to severe asthma, the amount of circulating ILC2 was increased in females compared to males, and in mice, IL‐2‐mediated ILC2 proliferation was increased in adult females compared to adult males and prepubertal females and males. The team ultimately concluded from their study that testosterone and androgen signalling attenuated IL‐2‐R‐mediated ILC proliferation, the number of ILC2 in the lungs, and ILC‐2‐mediated airway inflammation.^76^ Therefore, ILC2s play an important role in sex hormones and neurological actions in asthma, but the understanding of the mechanism of action of ILC2s in the immune response is still in its infancy, and the mechanisms of regulation and effects of ILC2s on allergic diseases still require more in‐depth exploratory studies.

#### 4.3.2 Peroxisome proliferator‐activated receptor γ

PPAR was discovered in rodents more than 30 years ago and was a subfamily belonging to the super family of ligand‐activated transcription factors nuclear hormone receptors.^77^ It is divided into three types, of which PPARγ is widely distributed in human nasal mucosa and nasal polyp mucosa and plays a role in inflammation by affecting the function of ILC2s through pathways such as acting on the tumour suppressor receptor on ILC2s (ST2), programmed cell death protein‐1 (PD‐1), and affecting the energy metabolism of ILC2s. Peroxisome proliferator‐activated receptor γ promotes liposorption and temporary lipid storage in lipid droplets, which are necessary to fuel the pathogenic ILC2 reaction that occurs during airway inflammation.^78^ In the review by Wang and colleagues, he summarised the relationship between PPARγ and ILC2. Peroxisome proliferator‐activated receptor γ may affect the metabolism of ILC2s through the pathways of ILC2s surface substances, PD‐1, and lipid metabolism. Furthermore, the metabolic sensor PPARγ is highly expressed in ILC2 in lung and adipose tissue. Moreover, pharmacological inhibition of PPARγ leads to reduced expression and fatty acid uptake of CD36, which is an essential energy source for ILC2 and potential ligands for PPARγ. The research team treated mice with PPARγ antagonists and found that it reduced the severity of ILC2‐dependent acute airway inflammation.^79^ PPARγ deficiency significantly impaired the ILC2 response to IL‐33, alleviating inflammatory disease of the airways. Further studies reported that the IL‐33 receptor ST2 guided the effect of PPARγ on ILC2. Qiang Xiao and others identified PPARγ as a key regulator of ILC2 in the lungs, which provides a renewed perspective on the effect of PPARγ in asthma.^80^

Hiromi Sato and colleagues conducted a study to evaluate the effects of sex hormones on PPAR expression and onset of activity in adipocyte cells. They added the sex hormones 17a‐oestradiol (E2), testosterone (T) or dihydrotestosterone (DHT) to mature adipocytes in mice. The results showed that longer E2 exposure dramatically increased the expression of PPARγ protein, while DHT exposure dramatically decreased its expression.^81^ This suggests that oestrogen may promote the expression of PPARγ protein, which in turn may have a role in airway inflammation such as asthma and AR by affecting ILC2s, further revealing a new pathway of action of oestrogen in triggering asthma. It has also been shown that PPARγ inhibits inflammatory processes in the CNS and reduces brain damage, improving motor and cognitive outcomes. PPAR‐γ lacking neurons are more susceptible to oxygenated glucose deprivation (OGD)‐caused damage.^82^, ^83^ Activation of PPARγ can have neuroprotective effects, reducing the inhibitory effects of the brain‐derived neurotrophic factor (BDNF) signalling pathway and acting by enhancing functions such as learning and memory, neurogenesis, and synapse formation.^84^

#### 4.3.3 NLRP3 inflammatory vesicles

The NLRP3 inflammasome, a multimeric cytosolic protein complex, is an essential component of the innate immune system, which is assembled and activated by NLRs in the pattern recognition receptor family that sense pathogen‐associated and damage‐associated molecular patterns, thereby Inducing the production of pro‐inflammatory factors.^85^ It is now well established that NLRP3 leads to activation of IL‐1‐produced inflammasome vesicles critical for the promotion of the Th2 inflammatory allergic reaction.^86^ It has been reported that oestrogen makes an important contribution by affecting NLRP3, and that oestrogen can inhibit the development of multiple diseases such as asthma, neurological disorders, asthma, bone disease, cancer etc. by suppressing NLRP3 inflammatory vesicles, but it can also promote disease progression by facilitating the activation of inflammatory vesicles.^87^ Therefore, further studies are required to investigate the molecular pathways involved in the dual effects of oestrogen on the development of asthma. NLRP3 also has some effects on the nervous system. Recent studies have shown that NLRP3 is a parkin substrate that drives neurodegeneration in Parkinson's disease. Loss of pachykinetic activity in mouse and human dopamine (DA) neurons leads to the assembly of NLRP3 inflammatory clusters, resulting in the death of DA neurons. In both familial and sporadic Parkinson's disease(PD) models, restraining of neuronal NLRP3 inflammatory cluster assembly could prevent the denervation of dopamine neurons.^88^ Therefore, the strategy of restricting neuronal NLRP3 inflammatory cluster activation holds potential as a therapeutic approach to disease modification for the treatment of neurological symptoms. Additionally, oestrogen, by inhibiting NLRP3 inflammatory vesicles, can also alleviate certain neurological symptoms such as cerebral ischaemia, MS, AD, PD and other disease symptoms.^89^

## 5 SUMMARY AND OUTLOOK

Asthma is currently one of the most widespread respiratory diseases, which seriously affects the life of patients and even has a certain impact on their psychology. With the in‐depth study of the respective roles of the nervous system and sex hormones on asthma pathogenesis, it was found that ILC2s, PPARγ and NLRP3 inflammatory vesicles play an important role in the pathways of action of the neurological system and sex hormones in influencing asthma pathogenesis. Sex hormones and the nervous system can influence the onset of asthma by acting on ILC2s, which means that in the future, for the study of the inhibition of ILC2s on asthma may be able to weaken the effects of the nervous system and sex hormones on asthma to a certain extent. And also, in asthma, PPARγ plays a positive regulatory role on the accumulation of ILC2s as well as in the functioning of effector, and sex hormones can promote the expression of PPARγ protein expression and triggers asthma, which provides new clues for asthma treatment and important guidelines for the development of the disease, as well as providing new targets for disease treatment, but the negative effects of neurological damage caused by PPAR inhibition should not be ignored, and further exploration and consideration are needed. In response to NLRP3 inflammatory vesicles, current research suggests that oestrogen can both inhibit NLRP3 to alleviate inflammation and activate NLRP3 to promote inflammation. In addition, oestrogen may slow certain neurological symptoms by inhibiting NLRP3 inflammatory vesicles. Therefore, how oestrogen can inhibit the NLRP3 inflammasome to exert protective effects or promote the involvement of the NLRP3 inflammasome in the development of disease, especially the former, requires further investigation. With the in‐depth study of the effects of oestrogen on NLRP3 inflammasome and the effects of NLRP3 inflammasome on the nervous system, NLRP3 inflammasome may be a potential target for the treatment of inflammatory diseases, which needs to be further investigated.

In addition, we have found that asthma is a very heterogeneous disease that is categorized into multiple phenotypes and endotypes to help address the complexity of the disease. The phenotypes of asthma include allergic asthma, delayed‐onset asthma, exercise‐induced asthma, and obesity‐associated asthma, and the endotypes of asthma include Th2‐high asthma, Th2‐low asthma, mixed‐inflammatory asthma, Paucigranulocytic asthma, and others.^90^ Allergic asthma is the most common asthma phenotype. It is a TH2‐driven process and is distinguishable by biomarkers such as serum IgE levels.^91^ In typical Th2‐high phenotype asthma, airway epithelium interacts with environmental factors to induce innate and adaptive immune and inflammatory responses, releasing TSLP, IL‐33, and IL‐25. The production of Th2 cells and ILC2 leads to the production of IL‐4, IL‐5, and IL‐13, which drives IgE production and recruitment of eosinophils to the lungs, resulting in multiple immune cell structural changes and activation of multiple immune cell types. Most immune cells express oestrogen receptors in varying degrees and are capable of responding to the hormone. Many studies have also shown that oestrogen levels correlate with symptom severity, likely contributing to gender differences in this type of allergic asthma.^92^ In the nervous system, sympathetic, parasympathetic and sensory nerves act on ILC2 by releasing neurotransmitters and thus promote or inhibit Th2‐type inflammatory responses, including Th2‐high asthma. Since this type of asthma is the most common type, in this paper we focus on studying and discussing the effects of sex hormones and the nervous system on the development of Th2‐high asthma.

In addition to Th2 high asthma, there are also Th2‐low asthma, mixed Th1/Th17 asthma, and Paucigranulocytic asthma. Th2‐low asthma is not associated with a type 2 inflammatory response and is usually present with asthma‐related comorbidities such as obesity and smoking. Obesity‐associated asthma is a non‐allergic severe phenotype that manifests as a loss of distal lung compliance associated with weight gain, some of the features of which are elevated airway hyperresponsiveness, oxidative stress, and so on. NLRP3, IL‐1β, and ILC3 cells also seem to play a role in obesity‐associated asthma and contribute to airway hyperresponsiveness associated with obesity‐associated asthma. Anti‐IL‐1β counteracts this effect by inhibiting IL‐17‐producing ILC3.^93^, ^94^ NLRP3 vesicles, as described above in this paper, can be inhibited by oestrogens to inhibit the development of certain diseases. Targeting NLRP3 vesicles may not only be important for Th2‐high asthma but may also have some positive significance for obesity‐related asthma phenotype. However, it is currently understudied and is a direction that needs to be explored with emphasis in the future. Similarly, it has been suggested that sex hormones may mediate the obesity asthma phenotype as a potential mediator.^95^ The association between obesity and asthma is stronger in female individuals than in male individuals.^96^ The use of oral hormonal contraceptives may increase the risk of asthma in obese women.^97^ These findings suggest that sex hormone differences may be a potential mechanism for obese asthma. In addition, Binaya Wasti and her team found that sex hormones, especially androgens, can inhibit Th17 cell differentiation by targeting Methyl‐CpG‐binding domain protein 2 (MBD2), which could potentially play a novel hormonal therapeutic role in Th17 cell‐dominated neutropenic severe asthma.^98^, ^99^ MBD2 has been found to be associated with neurodevelopmental and neurological disorders. Inhibition of MBD2 may inhibit the development of gliomas. This is because overexpression of MBD2 during gliomagenesis may inhibit the antiangiogenic activity of a key tumour suppressor, thereby promoting tumour growth.^100^ This also reveals the positive significance of MBD2 in linking both sex hormones and the nervous system in severe neutropenic asthma. It has also been shown that MBD2 deficiency may lead to the polarization of Th2 cells, which also triggered us to think about whether the phenotypes and endotypes can be interchanged between each other, and whether by acting on a target like MBD2 it could be helpful for treatment. The mechanisms of some other asthma phenotypes and endotypes, such as mixed Th1/Th2/Th17 asthma and Paucigranulocytic asthma, are not fully clarified, leading to their elevated therapeutic difficulty. There are also fewer studies related to the effects of sex hormones and the nervous system on these types of asthma attacks, which need to be further explored, which may guide us in discovering new therapeutic agents to provide better treatment. Research on whether the endotypes and phenotypes can be converted to each other and their mechanisms is also necessary. In the future, it may be possible to find one or several targets to regulate the conversion and superposition between the endotypes and phenotypes of asthma, thus reducing the complexity of the disease, decreasing the difficulty of treatment, and improving the therapeutic efficacy.

## AUTHOR CONTRIBUTIONS

**Wenting Zhou**: Writing ‐ original draft; Writing ‐ review & editing. **Huan Chen**: Writing ‐ review & editing. **Xinyu Chen**: Writing ‐ review & editing. **Jing Gao**: Writing ‐ review & editing. **Wenting Ji**: Writing ‐ review & editing.

## CONFLICT OF INTEREST STATEMENT

The authors declare no conflicts of interest.

## CONSENT TO PARTICIPATE

Not applicable.

## PATIENT CONSENT FOR PUBLICATION

Not applicable.

## Data Availability

Data will be made available on request.
